# A Novel Hyaluronic Acid-Black Rice Anthocyanins Nanocomposite: Preparation, Characterization, and Its Xanthine Oxidase (XO)-Inhibiting Properties

**DOI:** 10.3389/fnut.2022.879354

**Published:** 2022-04-14

**Authors:** Ya Liu, Bangzhu Peng

**Affiliations:** College of Food Science and Technology, Huazhong Agricultural University, Wuhan, China

**Keywords:** uric acid, black rice anthocyanins, hyaluronic acid, xanthine oxidase, inhibition

## Abstract

To promote the normal metabolism of human uric acid, high-performance hyaluronic acid-black rice anthocyanins (HAA) nanocomposite particles were successfully prepared by a simple crosslinking method as a novel xanthine oxidase inhibitor. Its structure and properties were characterized by scanning electron microscopy (SEM), transmission electron microscopy (TEM), Fourier transform infrared spectrometry (FT-IR), and X-ray diffraction (XRD). SEM and TEM electron microscopy showed an obvious double-layer spherical structure with a particle size of ~298 nm. FT-IR and XRD analysis confirmed that black rice anthocyanins (ATC) had been successfully loaded into the hyaluronic acid (HA) structure. Nanocomposite particles (embedded form) showed higher stability in different environments than free black rice ATC (unembedded form). In addition, the preliminary study showed that the inhibition rate of the nanocomposite particles on Xanthine oxidase (XO) was increased by 40.08%. These results indicate that HAA nanocomposite particles can effectively improve black rice ATC's stability and activity, creating an ideal new material for inhibiting XO activity that has a broad application prospect.

## Introduction

Uric acid (C_5_H_4_N_4_O_3_) is the final product of the metabolism of endogenous purines and exogenous purines *in vivo* and is a heterocyclic organic compound (1). When the level of uric acid in the blood is too high, it will accumulate in the form of urate in joints, cartilage, and other body tissues, causing swelling and deformation of organ tissues, resulting in gout arthritis and eventually the development of hyperuricemia. Xanthine oxidase (XO) is an important enzyme for regulating uric acid synthesis and metabolism (2). It is a molybdate protease composed of two completely symmetrical subunits. The catalytic center includes a molybdenum center, two iron-sulfur centers, and a xanthine adenine dinucleotide. The molybdenum center is the key site for the catalytic production of uric acid. The drugs that inhibit the production of uric acid are mainly XO inhibitors, so they are also one of the most promising therapeutic targets. Allopurinol and other commonly used traditional therapeutic drugs greatly impact human health due to their long curative effect and strong side effects (3, 4). Therefore, it is important to search for high efficiency and low toxicity in nutritional regulatory factors.

Black rice is a medicinal, edible rice formed by the long-term cultivation of gramineous plant rice. It has a long cultivation history and is an ancient and valuable rice variety in China (5, 6). Modern medicine has confirmed that black rice has the effects of nourishing the yin and kidney, invigorating the spleen, warming the liver, improving eyesight, and promoting blood circulation (7). This is mainly ascribed to the rich anthocyanins (ATC) in black rice skin. In recent years, the physiological functions of black rice ATC, such as alleviating liver injury, lowering blood lipids, acting as an anticancer agent, controlling diabetes, and preventing myocardial injury (8, 9), have been widely studied, and related literature reports that ATC have a strong inhibitory effect on XO activity (10). However, the instability and low bioavailability of black rice ATC limit their application in food ingredients. Although ATC can be directly absorbed by intestinal epithelial cells (11), they are easily degraded by the digestive environment of the small intestine. They are usually transferred to the colon with a low absorption rate (12). Thus, there is a considerable demand to develop a technology that can effectively improve ATC's bioavailability and physiological activity.

Nanoembedding technology has improved ATC stability by embedding ATC from light, heat, pH, and other environmental effects (13, 14). Hyaluronic acid (HA) is a polysaccharide in the extracellular and loose connective tissue of mammalian bone marrow cells. It is an ideal carrier for preparing nanomaterials because of its good biocompatibility and biodegradability (15–17). The hydrophobic drugs doxorubicin and camptothecin were loaded into HA to improve the water solubility and bioavailability of the drug (18, 19). Therefore, nanomaterials combined with HA and black rice ATC could be developed into a new type of health care substance, effectively improving the low stability of black rice ATC and enhancing the inhibition of XO activity.

The commonly used method for synthesizing nanomaterials is mainly chemical modification (20). Still, this method is more complicated and usually involves the addition of organic solvents, which have certain hidden dangers to human health when applied to food ingredients. Therefore, based on the group and charge characteristics of HA and black rice ATC, a simple crosslinking method was used to prepare hyaluronic acid-black rice anthocyanins (HAA) nanocomposite particles (21). The method is simple and has higher safety than the conventional chemical modification method. The nanocomposite particles were characterized by a Malven Zetasizer Nano-ZS (Nano-ZS), scanning electron microscopy (SEM), transmission electron microscopy (TEM), Fourier transform infrared spectroscopy (FT-IR), and X-ray diffraction (XRD). Then, the stability, *in vitro* release, and *in vitro* simulated digestion were evaluated to determine the effectiveness and applicability of HAA. For the first time, synthetic HAA was used as an inhibitor to improve the inhibitory effect on XO. This paper could also provide a reference for the development of novel enzyme inhibitors related to nanotechnology and provide a theoretical basis for the research and application of inhibiting XO activity in the fields of medicine and functional food.

## Materials and Methods

### Materials

A black rice-derived mixture of anthocyanins with a purity >25% (mainly containing Cyanidin-3-glucoside) was obtained from Xi 'an Xiaocao Plant Technology Co., Ltd. (Xi 'an, China). Hyaluronic acid (HA) (purity >95%) was purchased from Henan Sanhua Biological Technology Co., Ltd. (China). Xanthine oxidase (Cat. NO. X1875, from bovine milk, ≥0.4 units/mg protein, 5U) and xanthine (purity ≥99%) were purchased from Sigma–Aldrich Co. Ltd. (St. Louis, USA). Other chemicals were all analytical grade and were purchased from Sinopharm Chemical Reagent Co., Ltd. (Shanghai, China). All experimental water was deionized water.

### Preparation of HAA Nanocomposite Particles

The HA/ATC mass ratio (8–12: 1), reaction pH (2.8–4.8), and stirring time (1–5 h) were considered as the experimental factors for the preparation of HAA nanocomposite particles. The nanocomposite particles were prepared according to a previously reported method (22) with slight modifications. HA (18 mg) was dissolved in 10 mL of deionized water and stirred continuously until a clear and transparent solution was obtained. Then, under the action of magnetic stirring, 2 mL black rice ATC solution was slowly added into HA solution with a syringe and mixed evenly. The pH was adjusted to 4.3 with dilute hydrochloric acid (1.0 M). After ultrasonic treatment for 5 min (power 200 W, frequency 59 kHz, working for 10 s, intermittent 5 s), magnetic stirring was performed for 3 h to obtain a uniform pink suspension. Finally, the suspension was dried in a vacuum freeze to obtain HAA nanocomposite particles. All the above experiments were carried out in a dark environment.

### Encapsulation Efficiency (EE) Calculation

Black rice ATC's EE (%) was determined using the methods described previously (14, 23). The nanocomposite particles were centrifuged at 8,000 rpm for 30 min, and the free black rice ATC content in the supernatant was determined by the pH differential method. The samples were diluted with pH 1.0 (0.025 M) and pH 4.5 (0.4 M) buffers, and the absorbance was determined with distilled water as a blank at 510 and 700 nm, respectively. The black rice ATC content was calculated by the equivalent of cyanidin-3-glucoside (C3G) according to the equation (1):

(1) $$\begin{array}{r} \text{c }=\;\frac{\left(\text{ApH}1.0-\text{ApH}4.5\right)\;\times \text{ Mw }\times \text{ DF }\times \;1000}{\text{Ma }\times \text{ L}} \end{array}$$

where ApH1.0 and ApH4.5 are the maximum absorbance of pH 1.0 and pH 4.5 buffer dilution samples, respectively. Mw is the molecular equivalent (449.2 g/mol) of C3G. DF is the dilution factor; Ma is the extinction coefficient (26,900 mol/L^*^cm); L is the optical diameter (1 cm); and 1,000 is the conversion factor.

The encapsulation efficiency of the black rice ATC was calculated according to the equation (2):

(2) $$\begin{array}{r} \text{EE(\%)}=\frac{\text{ATC0}-\text{ATCt}}{\text{ATC0}}\times 100 \end{array}$$

where ATC0 is the initial content of black rice ATC in the nanocomposite particles, and ATCt is the content of free black rice ATC in the supernatant.

### Characterization of HAA Nanocomposite Particles

The particle size, zeta potential, and polydispersity index (PDI) of HAA nanocomposite particles were analyzed using a Malven Zetasizer Nano-ZS (Nano-ZS) (Malvern Inst. Ltd., UK) at 25°C. Scanning electron microscopy (SEM) (SU8010, Hitachi Ltd., Japan) and transmission electron microscopy (TEM) (H-7650, Hitachi, Tokyo, Japan) were used to determine the surface morphology of the samples. X-ray diffractometry (XRD) (D8 Advance, Brucker, Karlsruhe, Germany) was used to scan and determine the structure and composition of the nanocomposite particles at room temperature. The FT-IR spectra of nanocomposite particles were determined by Fourier Transform Infrared Spectrometer (FT-IR) (IS50, USA) scanning in the range from 4,000 cm^−1^ to 400 cm^−1^.

### Stability Analysis

The nanocomposite particles were stored for 6 days at 25°C and protected from light under 0.1, 0.25, 0.5, and 1.0 mg/mL ascorbic acid (AA) conditions. Samples were taken every day to detect the retention rate of black rice ATC. Sampling and detecting the retention rate of black rice ATC at appropriate intervals at three conventional temperatures of 4°C (cooling temperature), 25°C (room temperature), and 40°C (accelerated heating) (13). The nanocomposite particles were exposed to light and stored at 25°C for 10 days, and the retention rate of black rice ATC was measured every other day. The retention rate of black rice ATC was calculated by the pH differential method according to the equation (3, 24):

(3) $$\begin{array}{r} \text{R(\%) }=\;\frac{\text{Ct}}{\text{C0}}\times 100 \end{array}$$

where R represents the black rice ATC retention rate (%) (defined as the percentage of ATC content change); Ct represents the black rice ATC content sampled at time t (mg/L); and C0 represents the initial black rice ATC content (mg/L).

### *In vitro* Simulation Analysis

The *in vitro* sustained release analysis of nanocomposite particles referred to a slightly modified version of the previous method (25). The lyophilized nanocomposite particle powder was suspended in phosphate-buffered saline (PBS) buffer (0.1 M, pH 7.4) to fully dissolve it, transferred to an 8–14 kDa dialysis bag, sealed and put into a vial containing 50 mL PBS buffer, and shaken at 100 rpm (37°C). Then, 3 mL of the fluid outside the dialysis bag was removed at a fixed point, and fresh buffer solution with the same temperature and amount was immediately added. The drug release amount and cumulative drug release percentage were calculated, and the sustained release curves of the nanocomposite particles were plotted *in vitro*.

The *in vitro* simulated digestion of nanocomposite particles was evaluated according to a previous method (26) with slight modifications. Simulated oral digestion: 1 mL of activated saliva (6.5 mg α-amylase and 0.5 mg CaCl_2_ dissolved in ultra-pure water at pH 6.75) was added to a conical flask containing a 10 mL sample and digested at 100 rpm for 10 min (37°C). Simulated gastric digestion: After 10 min of simulated oral digestion according to the above steps, the oral digestive juice was adjusted to pH 2.0 with 6 M HCl, and then 1 mL of activated gastric juice (0.3 g of pepsin dissolved in 0.1 M HCl at pH 2.0), mixed well, and then shaken for 2 h. Simulated intestinal digestion: After oral and gastric simulated digestion according to the above steps, the gastric digestive juice was adjusted to pH 7.5 with 0.9 M NaHCO_3_, and then 10 mL of activated intestinal juice was added (3.8 g of pig bile salt and 0.6 g of trypsin were dissolved in 0.1 M NaHCO_3_ at pH 7.5) and mixed well, followed by shaking for 2 h. The retention rate of black rice ATC at different stages of simulated digestion *in vitro* was determined according to the method described in section stability analysis.

### Inhibitory Activity of Nanocomposite Particles on XO *in vitro*

To compare and analyze the inhibitory activities of nanocomposite particles on XO *in vitro*, the inhibition experiment was carried out according to a previous report with slight modifications (27). Briefly, a series of mixtures consisting of a standard concentration of XO (0.012 U/mL), PBS buffer (0.05 M, pH 7.5), and the samples were incubated in a 25°C constant temperature water bath for 30 min. Then, 1 mL xanthine (0.2 mg/mL) was added to start the reaction. Then, the time/kinetics software UV–Visible spectrophotometer (U3010, Hitachi Ltd., Japan) was used to determine the absorbance of uric acid at 290 nm in the reaction system and to determine the catalytic activity of uric acid produced by XO in the presence of nanocomposite particles with different concentrations. Then, the relative inhibition rate of the sample to XO was calculated according to the equation (4):

(4) $$\begin{array}{r} \text{I(\%) }=\;\left(1-\frac{\text{B}}{\text{A}}\right)\times 100 \end{array}$$

where B represents the enzyme reaction activity in the presence of inhibitor, and A represents the enzyme reaction activity in the absence of inhibitor.

### Statistical Analysis

All data are expressed as the means ± standard deviations of triplicate determinations. One-way analysis of variance (ANOVA) and Duncan's test (*P* < 0.05) were performed by SPSS 26.0 (IBM Inc., Armonk, NY, USA) to compare the differences between groups. Origin 8.0 (Origin Lab Inc., Massachusetts, USA) was used to draw relevant charts.

## Results and Discussion

### Preparation and Characterization of HAA Nanocomposite Particles

#### Nano-ZS Analysis

HA and black rice ATC will interact when mixed. The Malven Zetasizer Nano-ZS was used to determine the influence of different factors on the particle size, zeta potential and PDI value of the prepared HAA nanocomposite particles. The effect of the mass ratio of HA to black rice ATC on HAA nanocomposite particles is shown in Supplementary Table 1. With the increase contents of HA, the particle size of HAA decreased first and then increased gradually, and the zeta potential showed a downward trend, indicating that a large proportion of HA did not conform to the basic characteristics of nanocomposite particles. This may be because: the ionic interactions between black rice ATC and HA would be strengthened to a certain extent with the increase contents of HA (14), resulting in a tighter combination of the two. Further increasing the HA content exceeded the binding limit with black rice ATC, too much water-soluble carrier attached to the surface of the nanocomposite particles or float around, leading to the gradual increase of the particle size. The effect of pH on the nanocomposite particles is shown in Supplementary Table 2. With the increase of pH, the particle size and PDI of the nanocomposite particles decreased relatively, and zeta potential relatively increased, but as the pH continued to increase, the zeta potential decreased. This may be because the small pH (pH < 3) increased the repulsive force between the positive charge density of black rice ATC and the -NH_2_ groups, which destabilized the polymer network and induced the swelling of the particles. The electrostatic force between the positive charge of black rice ATC and anionic polysaccharides at pH values of about 3 and 4 was strong, resulting in a close binding between the two. As the pH value continued to increase, the electrostatic force between HA and black rice ATC weakened, and PDI increased, resulting in condensation. The effect of stirring time on the nanocomposite particles is shown in Supplementary Table 3. With the increase of stirring time, the particle size of the nanocomposite particles gradually decreased and was evenly dispersed. However, as the stirring time continued to increase, the particle size and PDI value increased sharply. This may be due to the dynamic formation process of HAA nanocomposite particles. Appropriate stirring time was conducive to the dispersion of solutes and the uniform particle size of nanocomposite particles. Excessive stirring destroyed the interaction between HA and black rice ATC, increasing the frequency of collisions and aggregations in the system, thus affecting the morphology and homogeneity of the nanocomposite particles. Further experimental results and range analysis are shown in Supplementary Tables 4, 5, and the stirring time had the greatest influence on the particle size and zeta potential of HAA nanocomposite particles. Based on the analysis results of particle size and zeta potential, the stirring time 3 h, pH 4.3, and HA:ATC mass ratio 9:1 were considered optimal conditions for preparing HAA nanocomposite particles.

To further characterize the preparation conditions of the HAA nanocomposite particles, verification experiments were carried out, and the results are shown in Table 1. The prepared nanocomposite particles showed a suitable particle size. Generally, since 50–500 nm nanocomposites are optimal for epithelial cells (28), HAA nanocomposite particles are favorable for absorption by intestinal epithelial cells. In addition, the PDI value of the nanocomposite particles prepared under the process conditions was acceptable. The zeta potential value can reflect nanoparticles' physical and chemical characteristics and biological stability in solution. The zeta value of the nanocomposite particles prepared in this experiment was larger, indicating that the state of the nanoparticles in the system was relatively stable. Moreover, the higher encapsulation efficiency is conducive to improving the utilization of small molecule active substances. It is speculated that the electrostatic attraction between the black rice ATC cation and the carboxyl groups on HA would enhance the encapsulation of black rice ATC. It may also be because the dense network formed by hydrogen bonds between black rice ATC and HA contributed to the improvement of embedding efficiency, which has a better protective effect on black rice ATC (24).

**Table 1:** Average particle size, zeta potential, PDI, and EE (%) of HAA nanocomposite particles.

**Samples**	**Particle size (nm)**	**zeta potential (mV)**	**PDI**	**EE (%)**
9:1-pH 4.3–3 h	298.56 ± 5.51	−32.51 ± 0.99	0.343 ± 0.02	86.04 ± 0.5

#### Morphological Analysis

**Figure 1:**
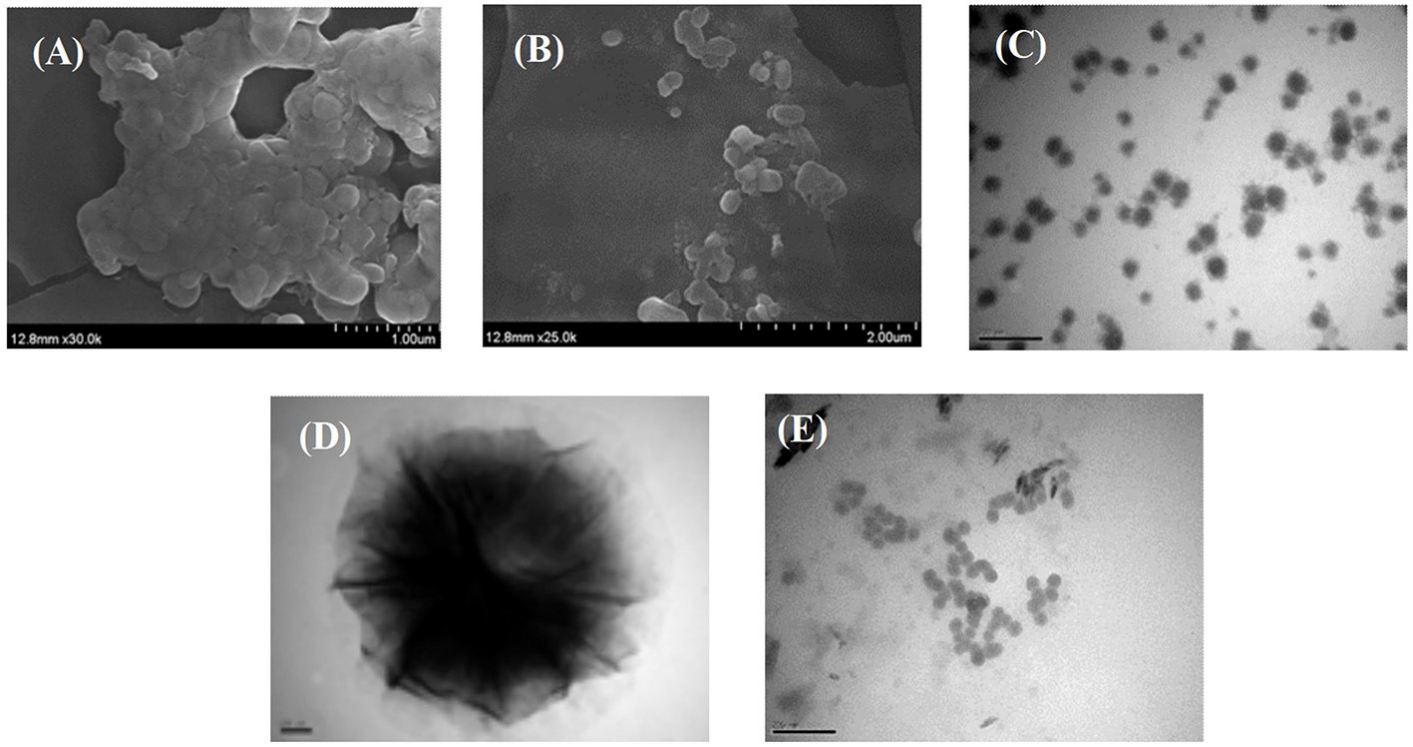
SEM images of HAA nanocomposite particles **(A,B)**, TEM images of HAA nanocomposite particles **(C,D)**, and HA **(E)**.

TEM and SEM were used to evaluate the morphology of HAA nanocomposite particles, which is helpful to explain the microstructure and aggregation characteristics of HAA. As shown in Figures 1A,B, the synthesized HAA nanocomposite particles had a clear structure without obvious agglomeration phenomena, which is similar to other biopolymer-based nanomaterials. As shown in Figures 1C,D, the nanocomposite particles were evenly dispersed, with a single spherical shape and an average particle size of 200 nm. However, it should be noted that there was a difference between the particle size measured by the Malvern particle size analyzer and the transmission electron microscope, which may be due to the loss of water during freeze-drying. In general, the average particle size determined by the Malvern particle size analyzer is closer to the size of the nanoparticle in the systemic circulation system, which is more suitable for the absorption and utilization of the biological environment (29). In addition, compared with HA alone (Figure 1E), an obvious double-layer structure could be found after the nanocomposite particles formed, which could intuitively reflect that the black rice ATC had been successfully loaded into the network structure of HA.

### Structural Analysis

**Figure 2:**
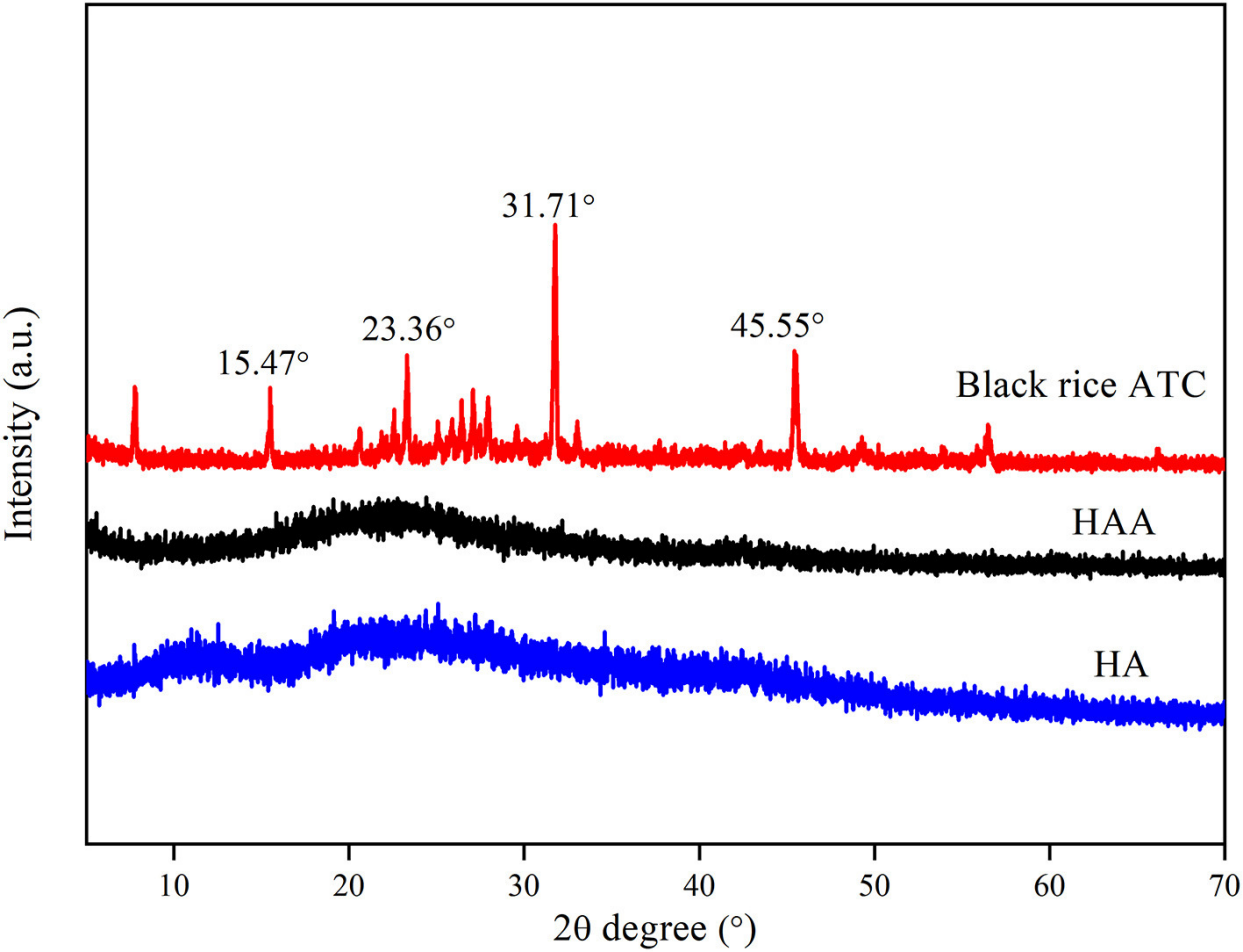
XRD spectra of HA, black rice ATC, and HAA nanocomposite particles.

As shown in Figure 2, the diffraction peaks of black rice ATC were mostly sharp, and characteristic peaks were observed at 2θ values of 15.47°, 23.36°, 31.71°, and 45.55°, which indicated that black rice ATC was highly crystalline. Two relatively wide diffraction peaks were detected at 2θ values of 11.31° and 19.73°, indicating that HA was semicrystalline. The diffraction peak intensity of the synthesized nanocomposite particles was significantly reduced, showing the characteristics of amorphous polymers. The results showed that the crystal structure of the black rice ATC molecule in the nanocomposite particles was covered, and that the diffraction peak intensity was significantly reduced, which may be related to the newly formed intermolecular hydrogen bond between black rice ATC and HA.

### Spectral Analysis

**Figure 3:**
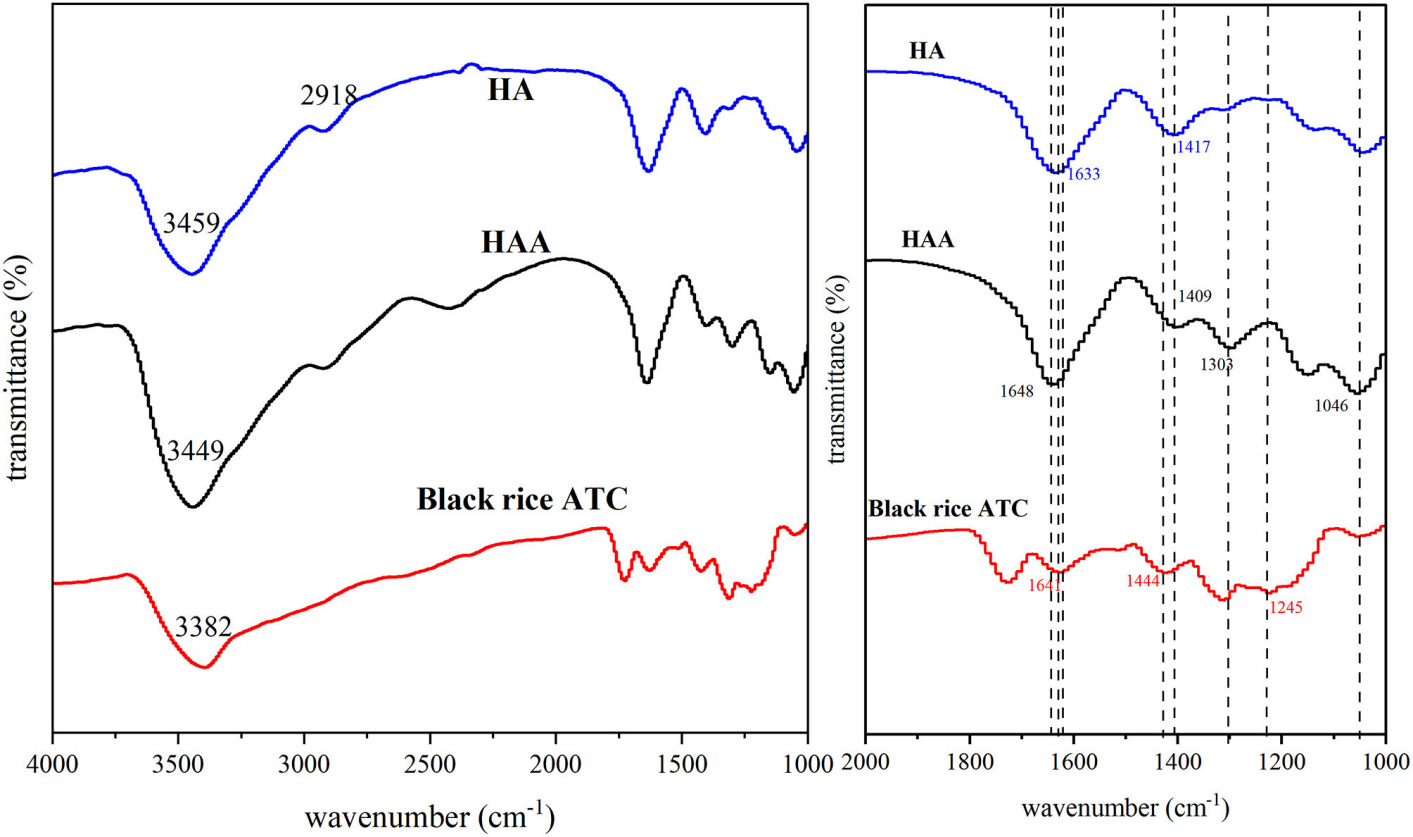
The averaged and normalized FT-IR spectra of HA, black rice ATC, and HAA nanocomposite particles.

The averaged and smoothed FT-IR spectra of HA, black rice ATC, and HAA nanocomposite particles are shown in Figure 3, and the characteristic peak positions of HA, black rice ATC and HAA are shown in Supplementary Table 6 (30, 31). The positions of the characteristic peaks were determined according to previously methods (23, 32, 33). The peaks of black rice ATC at 1,641 and 1,444 cm^−1^ corresponded to the stretching vibrations of C=C and C=O in the aromatic ring skeleton, respectively. A band at 1,326 cm^−1^ corresponded to C-O angular deformations of phenols (13), and 1,245 cm^−1^ was the stretching vibration peak caused by the benzopyran aromatic ring, which was a typical flavonoid structure peak. The peaks of HA at 1,633 and 1,417 cm^−1^ belonged to the -COO^−^ group of carboxylic acid salt. In the spectral bands of the synthesized nanocomposite particles, the stretching vibration of C=O (1,648 cm^−1^) showed a blueshift absorption peak, and the stronger stretching vibration at 1,303 cm^−1^ was attributed to the introduction of black rice ATC, while the absorption peak at 1,046 cm^−1^ was stronger than that of HA, indicating hydrogen bond formation. Moreover, there was a shift in O-H stretching vibrations from 3,382 to 3,449 cm^−1^, revealing hydrogen bonding between black rice ATC and HA (34). The characteristic peak of ATC disappeared at 1,245 cm^−1^ in HAA, indicating that HA had successfully embedded black rice ATC. In addition, the COO^−^ characteristic peak of HA at 1,417 cm^−1^ was shifted to 1,409 cm^−1^ in HAA, indicating the electrostatic interaction between -COOH of HA and -OH of black rice ATC. The above results all confirmed the successful combination of HA and black rice ATC.

### Stability Analysis

Affected by its own structure, ATC are often unstable to the external environment and are easily degraded. The expectation that HA would stabilize black rice ATC by providing a barrier is supported by studies examining free black rice ATC and HAA storage. The stability analysis of free black rice ATC and HAA nanocomposite particles under different environmental stresses and storage conditions is shown below.

#### Effect of Ascorbic Acid Treatment

**Figure 4:**
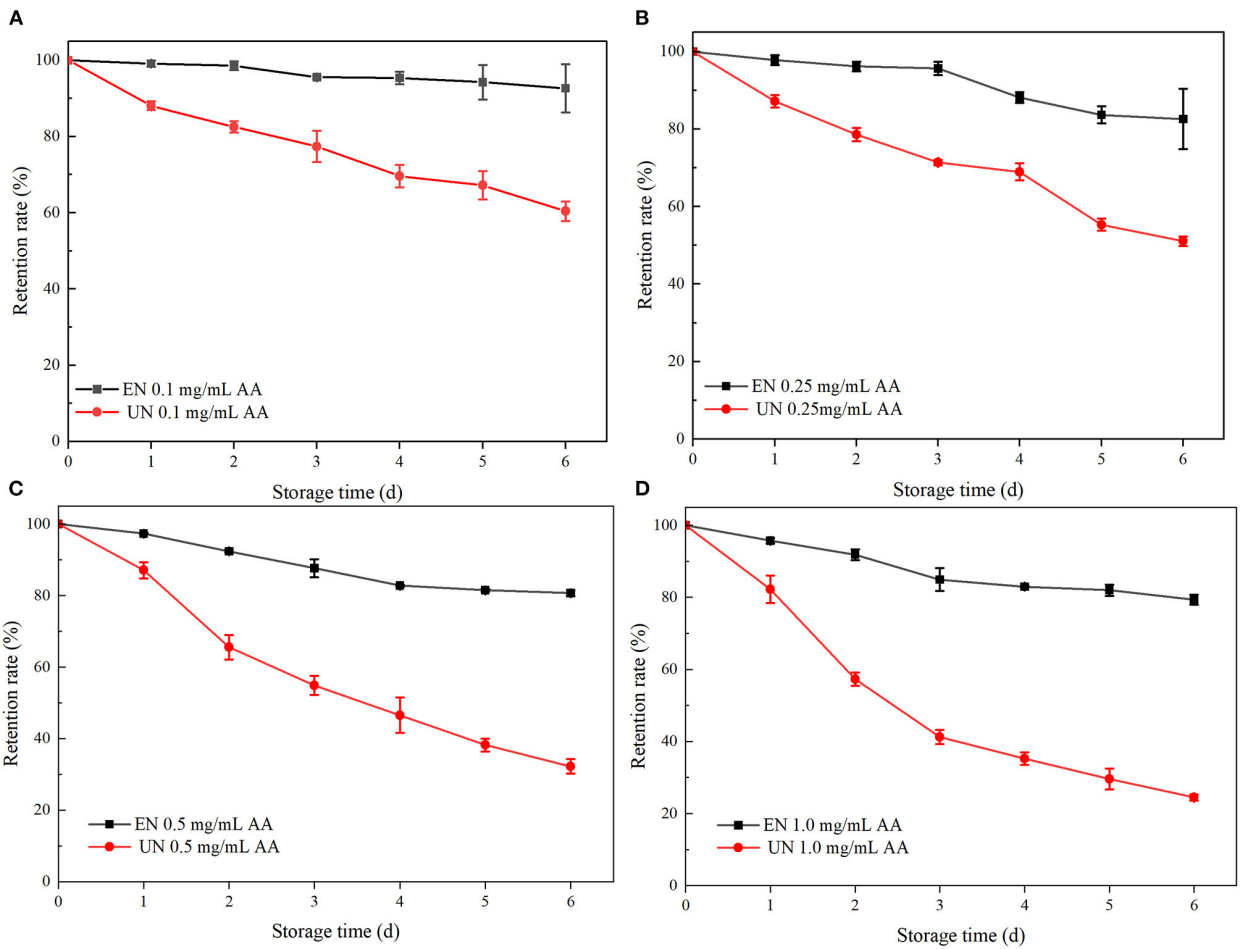
The stability of black rice ATC at different ascorbic acid (AA) concentrations [**(A)** 0.1 mg/mL AA, **(B)** 0.25 mg/mL AA, **(C)** 0.5 mg/mL AA, and **(D)** 1.0 mg/mL AA]. EN and UN represent HAA nanocomposite particles (embedded form) and black rice ATC (unembedded form), respectively.

AA is a common additive in food. Relevant studies have shown that a certain amount of AA has a degradation effect on ATC, mainly due to the effect of hydrogen peroxide (H_2_O_2_). H_2_O_2_ is an auto oxidizing product of AA that opens the ring of ATC and leads to the oxidation of flavonoid salts into colorless products, leading to ATC degradation (35–37). As shown in Figure 4, the retention rates of free black rice ATC at different AA concentrations gradually decreased with the extension of storage days, and the retention rates after 6 days were 60.39, 51.08, 32.24, and 24.49%, respectively. However, HAA nanocomposite particles showed significant protection against ATC degradation at the same AA concentration. The retention rates were increased by 32.27, 31.5, 48.42, and 54.84%, respectively, which was in accordance with previously reported studies (13). The encapsulation of nanocomposite particles provided a physical barrier to prevent direct contact between black rice ATC and AA, thus improving stability. The higher retention rate of HAA nanocomposite particles was also attributed to the higher encapsulation efficiency.

#### Effect of Thermal Treatment

**Figure 5:**
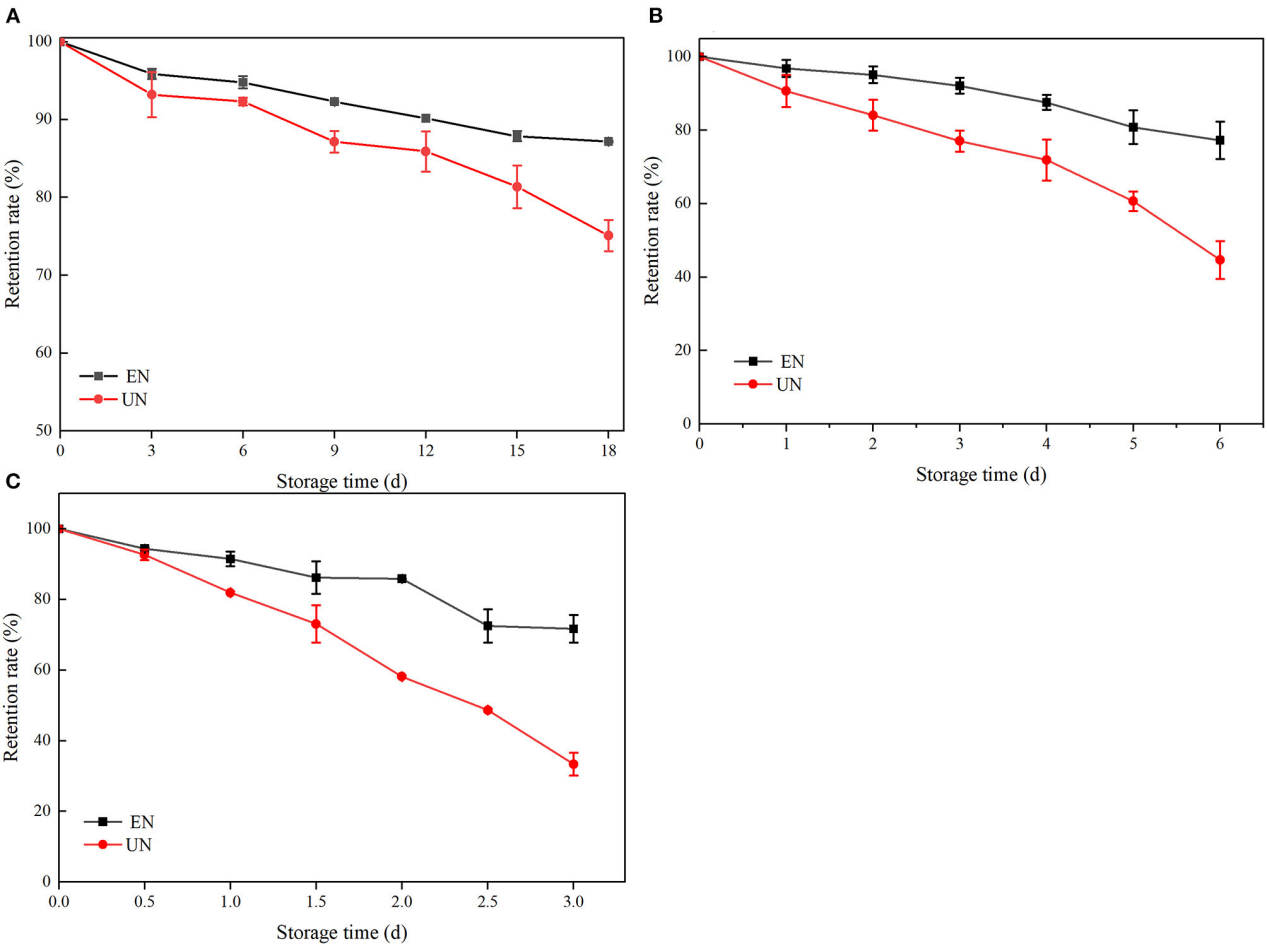
The stability of black rice ATC at different temperatures [**(A)** 4°C, **(B)** 25°C, and **(C)** 40°C].

ATC are sensitive to temperature. The stability analysis of free black rice ATC and HAA under different conventional temperatures is shown in Figure 5. The black rice ATC retention rates of HAA nanocomposite particles stored at 4°C for 18 days, 25°C for 6 days, and 40°C for 3 days were 87.18, 73.68, and 71.71%, respectively, while the free black rice ATC stored at 4, 25, and 40°C for the same time were only 75.09, 44.68, and 33.37%, respectively. The HAA nanocomposite particles showed better thermal stability than free black rice ATC did. After the interaction of HA with black rice ATC, black rice ATC was not reduced to a colorless chalcone structure and methanol pseudoalkaline form, which prevented the hydration of black rice ATC and thus maintained thermal stability. Generally, water-soluble carbohydrates can significantly improve the thermal stability of ATC by reducing the water activity around ATC (38). Our experimental results show that the nanocomposite particles can effectively reduce the degradation of black rice ATC caused by temperature factors, providing new insight for improving the stability of black rice ATC at different storage temperatures and expanding its wide application in food.

### Effect of Light Treatment

**Figure 6:**
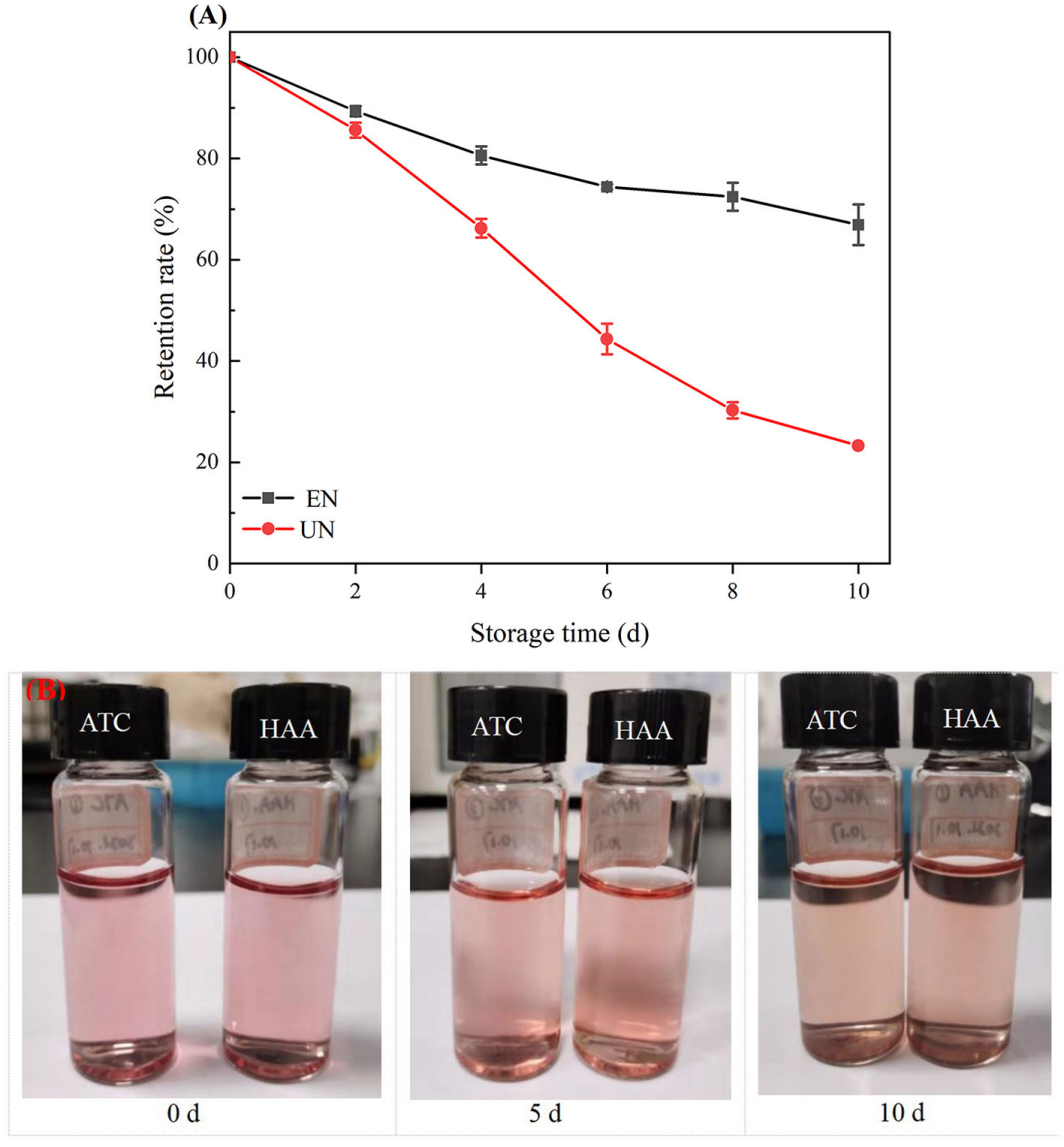
**(A)** The stability of black rice ATC under 10 days of light; **(B)** Photographs of free ATC and HAA under 10 days of light.

Light is an important factor affecting the degradation of ATC. As shown in Figure 6A, after 10 days of light treatment, the retention rate of free black rice ATC was 23.28%, while the retention rate of HAA nanocomposite particles increased by 43.63%, showing an obvious protective effect. ATC are prone to discoloration and degradation during exposure to light (39). As shown in Figure 6B, with the prolongation of light, the color of free black rice ATC changed from pink to light yellow and finally to dark yellow. In contrast, the color change of HAA nanoparticles was less obvious, indicating that HAA effectively protected the degradation of black rice ATC under light and improved its stability. The color changes of free black rice ATC and HAA nanocomposite particles were consistent with the change trend of black rice ATC retention rate, which indicated that HA embedding can effectively reduce the degradation of black rice ATC and improve the light stability.

The above conclusion could be explained from the strong interaction between HA and black rice ATC observed in XRD and FT-IR spectra. We believe that the stability of the HAA nanocomposite particles is due to the high permeability barrier of the HA carrier in the outer layer to polar oxidants (such as ascorbic acid) and degradation under heat and light.

### *In vitro* Simulation Analysis

#### Analysis of Sustained Release *in vitro*

**Figure 7:**
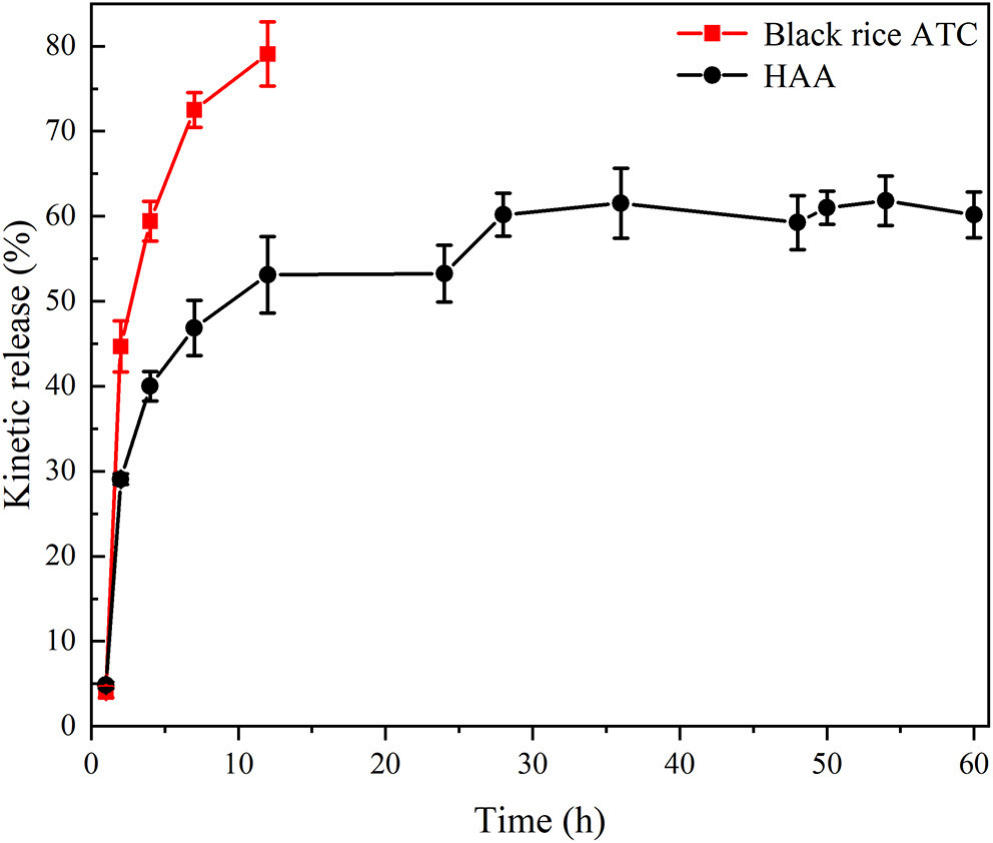
*In vitro* sustained-release diagram of black rice ATC and HAA nanocomposite particles.

The release characteristics of HAA nanocomposite particles embedded with black rice ATC are not only affected by gastrointestinal environment, but also related to the encapsulation material. Before simulating gastrointestinal digestive environment, exploring *in vitro* release characteristics of HAA nanocomposite particles embedded with HA is crucial to understand whether the nanocomposite particles can play an active role *in vivo* and *in vitro*, which can reflect the release of bioactive substances in the human digestive tract. As shown in Figure 7, free black rice ATC was released rapidly in the first 12 h and reached 60% at 4 h, showing sudden release behavior. In comparison, the release rate of HAA was faster in the first 12 h and then leveled off, and the total release reached 60% after 60 h. Therefore, the nanocomposite particles showed significant *in vitro* sustained release characteristics. This phenomenon may be that the interaction force between black rice ATC and HA made free black rice ATC not easy to diffuse, or the protective layer after HA embedded black rice ATC delayed the release of ATC. Similar studies have also pointed out that sustained release can reduce the degradation of active compounds, thus increasing the absorption and bioavailability of more active compounds in the gastrointestinal tract (40).

#### Analysis of Simulated Digestion *in vitro*

**Figure 8:**
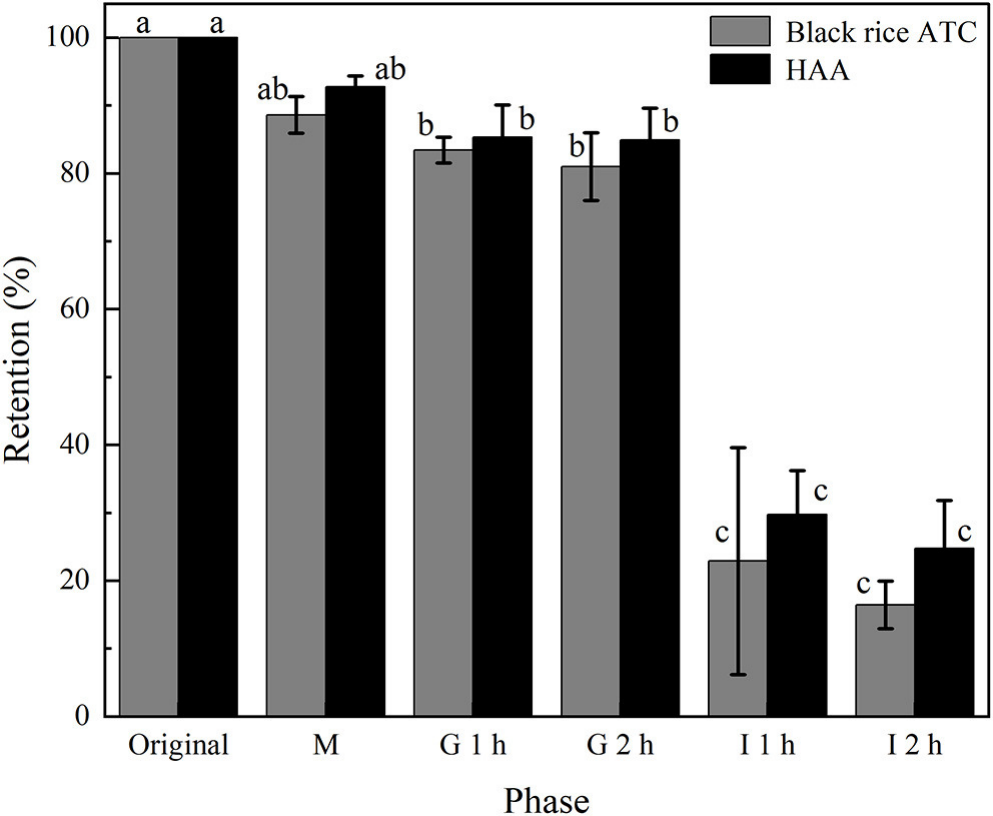
ATC content changes of free black rice ATC and HAA nanocomposite particles during simulated digestion *in vitro*.

Based on the sustained-release characteristics of HAA nanoparticles *in vitro*, the effect of the simulated gastrointestinal digestive environment after the release of the HAA nanocomposite particles was further analyzed (Figure 8).

In the digestion process, the bioavailability of ATC will be affected due to factors such as pH value and digestive enzymes. The stability of the HAA nanocomposite particles and free black rice ATC was evaluated during the simulated digestion process *in vitro*. As a whole, the black rice ATC content was not significantly affected by oral and gastric simulated digestion environments, which was similar to the effect of *in vitro* simulated digestion on ATC content from other extracts (14) and was also related to the better stability of ATC under acidic conditions (41). In addition, oral digestive enzymes and gastric digestive enzymes did not exert significant metabolic activity on ATC (42). In contrast, simulated intestinal digestion resulted in a sharp decrease in the ATC content of free black rice ATC and HAA nanocomposite particles. After 2 h of intestinal digestion, the ATC content of free black rice ATC and nanocomposite particles decreased to 16.46 and 24.73%, respectively. This phenomenon may be related to the effect of the intestinal pH value on the structural integrity of black rice ATC. Also, bile salts in intestinal juice gradually replaced the polysaccharides adsorbed on the surface, destroying the structure of HAA, and rapidly degrading black rice ATC (43). The retention rate of black rice ATC in HAA nanocomposite particles was always higher than that of free black rice ATC during the whole simulated digestion process *in vitro*, which may be because the black rice ATC was embedded in HA, which modified the degradation of black rice ATC in the simulated gastrointestinal tract. The results showed that nanoembedding technology could reduce the degradation of active compounds and improve the bioavailability of more active compounds in gastrointestinal digestion and absorption. However, the actual human digestive process is much more complicated, and its digestion and absorption mechanisms still need further study.

### Evaluation of XO Activity *in vitro*

**Figure 9:**
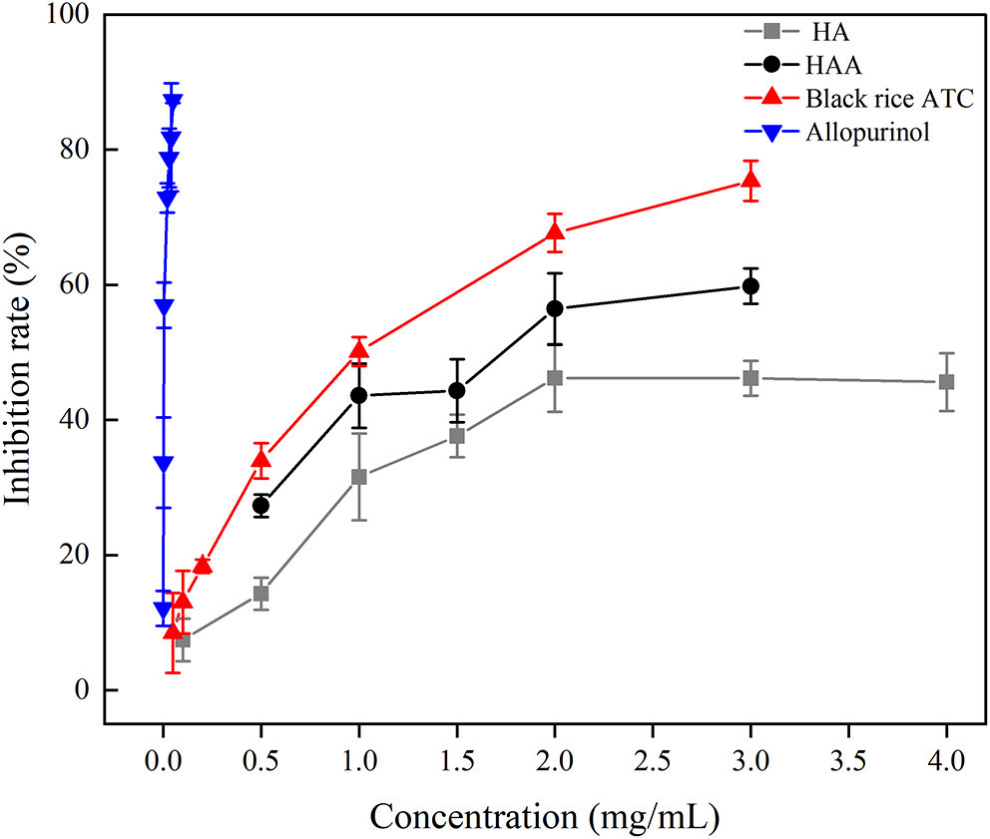
The inhibitory effect of black rice ATC, HA, and HAA nanocomposite particles on XO.

XO is a key enzyme that produces uric acid in the human body and is a common drug target. Inhibiting XO activity becomes the main way to reduce the production of uric acid. ATC has a strong inhibitory effect on XO activity *in vitro* (10). To further test whether the synthesized HAA nanocomposite particles improved the inhibitory effect on XO activity, the activity of producing uric acid catalyzed by XO in the reaction system was detected *in vitro*. According to the mass ratio of HA and black rice ATC of 9:1, the inhibitory effects on XO activity were detected, and the results are shown in Figure 9. The *in vitro* inhibitory rate of black rice ATC (0.2 mg/mL) was 16.34%, indicating that black rice ATC had a certain inhibitory effect on XO. The inhibitory rate of HA (1.8 mg/mL) on XO was 40%, indicating that HA also had a certain inhibitory effect on XO. When HAA nanocomposite particles were used as inhibitors to inhibit XO activity, the inhibitory rate of HAA nanocomposite particles (2.0 mg/mL) on XO activity *in vitro* reached 56.42%, the inhibitory effect improved with increasing concentration. HA and black rice ATC have a synergistic effect of inhibiting XO activity effectively. Although the inhibitory effect of HAA on XO was not as strong as that of allopurinol, our results suggested that HAA is a promising XO inhibitor. In addition, many studies have found that allopurinol has strong toxicity and side effects, which can cause symptoms such as inflammatory cell infiltration of the renal interstitium and renal duct dilatation. The above results confirmed the effectiveness and applicability of HAA nanocomposite particles prepared by a simple cross-linking method in inhibiting XO, which provided a new idea for inhibiting the production of uric acid.

## Discussion

The pH effect and molecular groups during simple crosslinking are a reasonable explanation for the preparation of HAA nanocomposite particles. Hyaluronic acid is a unique negatively charged acidic mucopolysaccharide, which differs from other acidic mucopolysaccharides in that the acid group is carboxyl instead of sulfuric acid (44). The surface of ATC is positively charged at pH 3 and 4 (45), and the presence of carboxyl functional groups in polysaccharides ensures strong electrostatic interaction and hydrogen bonding with ATC. The particle size of the nanocomposite particles increased with the increase of polysaccharide (Supplementary Table 1). With the increase of HA, too much water-soluble carrier adhered and aggregated, resulting in a gradual increase of the particle size. In addition, the free NH^3+^ in the polysaccharide structure increased, resulting in greater repulsion and larger diameter of nanoparticles (46). The surface of ATC is positively charged when the pH value is about 3 and 4, and the electrostatic force closely bound with HA. The electrostatic force weakened as the pH increased, resulting in intermolecular repulsion, particle size dispersion and agglomeration (Supplementary Table 2). The interaction between HA and black rice ATC groups was well-reflected in XRD and FT-IR spectra. The intensity of the characteristic absorption peaks of HA and black rice ATC and the changes of functional groups are attributed to the combination between the phenolic hydroxyl group of black rice ATC and COO- on HA, resulting in hydrogen bond formation and electrostatic force generation, which makes the formation of black rice ATC load HA nanocomposites possible. However, the structural interaction form between HA and black rice ATC and the cross-linking mechanism still need to be further studied in the future.

ATC are easily affected by external environment and undergo degradation, which affects bioavailability. HA improved the environmental stress and storage stability of black rice ATC by providing a covering layer and weakening environmental damage, and significantly inhibited AA, heat, and light-induced degradation of ATC. This also benefits from the stabilizing force formed between HA and black rice ATC. Therefore, HA is a good candidate material to study the inhibition of black rice ATC degradation. This study improves the stability of black rice ATC in different environments and expands their wide application in food.

ATC are also easily degraded *in vivo*, and their bioavailability *in vivo* is affected not only by gastrointestinal digestive environment, but also by encapsulation materials (40). Some encapsulation materials can affect the utilization of encapsulated active compounds *in vivo*, affect their effective release ability in the gastrointestinal tract, and limit their absorption (47). The nanocomposite particles formed by HA-encapsulated black rice ATC had significant slow-release properties, which would not affect the release of black rice ATC, but also reduced the effect of black rice ATC on the environment *in vivo* and avoided rapid degradation. Excluding the effect of embedding materials, free black rice ATC were rapidly degraded during the entire digestion process, and HAA nanocomposite particles significantly reduced the effect of black rice ATC on gastrointestinal digestive environment due to their good barrier ability, confirming the embedding protective effect of nanocomposites. This conclusion also confirmed the previous results that ATC could improve the stability of the external environment. In conclusion, the encapsulation of nanocomposite particles not only enhances the stability of black rice ATC *in vitro*, but also improves its absorption and bioavailability *in vivo* to a certain extent, indicating that the biological function of black rice ATC may be enhanced. Compared with free black rice ATC, HAA nanoparticles had a synergistic effect on XO *in vitro*. However, the binding characteristics of HA and black rice ATC synergistically inhibiting XO and their respective contributions to XO need further study. Moreover, the scope and limitations of the synergistic effect of HA and ATC still need to be further studied.

## Conclusions

This study proposed a novel and efficient simple cross-linking method to prepare HAA nanocomposite particles to inhibit XO activity. Compared with the conventional chemical modification method, this method is simple to operate and does not require the addition of any organic solvents. The introduction of HA enhanced the stability of black rice ATC under different environmental stress (AA) and storage conditions (thermal and light). It delayed the release of black rice ATC *in vitro* (the total release amount of black rice ATC reached 60% at 60 h, with significant, sustained-release characteristics). The bioavailability of active compounds in *in vitro* digestion and absorption was improved. In addition, compared with free black rice ATC, the inhibition rate of XO activity was increased by 40.08%. This discovery indicates that HAA nanocomposite particles have the potential to stabilize and enhance the physiological activity of black rice ATC, which is of great significance for the development of novel nanocomposite materials for inhibiting XO activity. However, in this study, the preparation and characterization of the nanocomposite particles and the inhibition of XO were verified *in vitro*. Whether the black rice ATC and the HAA nanocomposite particles can play a good role in inhibiting XO activity and lowering uric acid *in vivo* remains to be further determined, which needs to be analyzed by animal experiments in later experiments.

## Data Availability Statement

The original contributions presented in the study are included in the article/Supplementary Material, further inquiries can be directed to the corresponding authors.

## Author Contributions

YL: conceptualization, methodology, data curation, formal analysis, writing—original draft, and writing—review and editing. BP: resources, writing—original draft, writing—review and editing, supervision, project administration, and funding acquisition. All authors contributed to the article and approved the submitted version.

## Funding

This research was financed by the National Natural Science Foundation of China (Grant No. 31972063).

## Conflict of Interest

The authors declare that the research was conducted in the absence of any commercial or financial relationships that could be construed as a potential conflict of interest.

## Publisher's Note

All claims expressed in this article are solely those of the authors and do not necessarily represent those of their affiliated organizations, or those of the publisher, the editors and the reviewers. Any product that may be evaluated in this article, or claim that may be made by its manufacturer, is not guaranteed or endorsed by the publisher.
