# Supplementary material for: A Novel Hyaluronic Acid-Black Rice Anthocyanins Nanocomposite: Preparation, Characterization, and Its Xanthine Oxidase (XO)-Inhibiting Properties
Source: Front Nutr. 2022 Apr 14;9:879354. doi: 10.3389/fnut.2022.879354 (PMC9048741; doi:10.3389/fnut.2022.879354)
Supplement: Supplementary file 1 [file Data_Sheet_1.docx]

Supplementary Material

# Supplementary Figures and Tables

## Supplementary Tables

Table S1 Effect of HA:ATC mass ratio on HAA composite nanoparticles

| mass ratio | particle size (nm) | PDI | zeta potential (mV) |
| --- | --- | --- | --- |
| 8:1 | 192.96±67.83^bc^ | 0.41±0.085^ab^ | -28.65±2.15^c^ |
| 9:1 | 182.03±76.01^bc^ | 0.47±0.064^ab^ | -15.05±1.85^b^ |
| 10:1 | 122.33±5.44^c^ | 0.33±0.028^b^ | -27±2.59^c^ |
| 11:1 | 261.53±36.42^b^ | 0.55±0.047^a^ | -5.5±1.20^a^ |
| 12:1 | 524.9±9.40^a^ | 0.48±0.051^ab^ | -17.76±1.61^b^ |

Note: Different superscript letters in the same column indicate significant difference (P < 0.05), and the value is expressed as mean ±SD (n=3).

Table S2 Effect of pH value on HAA composite nanoparticles

| pH | particle size (nm) | PDI | zeta potential (mV) |
| --- | --- | --- | --- |
| 2.8 | 789.3±19.80^a^ | 0.58±0.013^a^ | -24.5±4.8^ab^ |
| 3.3 | 277.95±23.45^c^ | 0.43±0.015^ab^ | -24.8±1.0^ab^ |
| 3.8 | 622.55±5.15^b^ | 0.6±0.025^a^ | -20.6±0.7^a^ |
| 4.3 | 601.15±5.15^b^ | 0.36±0.024^b^ | -32.1±0.4^b^ |
| 4.8 | 280.45±3.85^c^ | 0.59±0.018^a^ | -18.8±0.6^a^ |

Note: Different superscript letters in the same column indicate significant difference (P < 0.05), and the value is expressed as mean ±SD (n=3).

Table S3 Effect of reaction stirring time on HAA composite nanoparticles

| time (h) | particle size (nm) | PDI | zeta potential (mV) |
| --- | --- | --- | --- |
| 1 | 350.56±39.68^a^ | 0.478±0.068^a^ | -20.9±1.9^a^ |
| 2 | 291.25±1.65^ab^ | 0.56±0.012^a^ | -24.55±1.15^ab^ |
| 3 | 279.05±49.25^ab^ | 0.51±0.051^a^ | -28.93±2.36^b^ |
| 4 | 187.2±7.1^b^ | 0.33±0.028^b^ | -24.75±0.15^ab^ |
| 5 | 312.95±5.65^a^ | 0.54±0.011^a^ | -29±4.34^b^ |

Note: Different superscript letters in the same column indicate significant difference (P < 0.05), and the value is expressed as mean ±SD (n=3).

Table S4 Average particle size orthogonal experiment results and range analysis

| number | A  mass ratio | B  pH | C  time（h） | particle size（nm） |
| --- | --- | --- | --- | --- |
| 1 | 8:1 | 3.3 | 2 | 846 |
| 2 | 8:1 | 3.8 | 3 | 399.5 |
| 3 | 8:1 | 4.3 | 4 | 391.7 |
| 4 | 9:1 | 3.3 | 3 | 216.4 |
| 5 | 9:1 | 3.8 | 4 | 257.7 |
| 6 | 9:1 | 4.3 | 2 | 183.6 |
| 7 | 10:1 | 3.3 | 4 | 273.8 |
| 8 | 10:1 | 3.8 | 2 | 1365 |
| 9 | 10:1 | 4.3 | 3 | 191 |
| Average 1 | 545.750 | 445.417 | 798.200 |  |
| Average 2 | 219.250 | 674.083 | 268.967 |  |
| Average 3 | 609.950 | 255.450 | 307.783 |  |
| R | 390.700 | 418.633 | 529.233 |  |
| Primary and secondary factors |  | C＞B＞A |  |  |
| Optimal solution |  | C2 B3A2 |  |  |

Table S5 Zeta potential orthogonal experiment results and range analysis

| number | A  mass ratio | B  pH | C  time（h） | zeta potential  （mV） |
| --- | --- | --- | --- | --- |
| 1 | 8:1 | 3.3 | 2 | -7.05 |
| 2 | 8:1 | 3.8 | 3 | -36.35 |
| 3 | 8:1 | 4.3 | 4 | -32.6 |
| 4 | 9:1 | 3.3 | 3 | -27.2 |
| 5 | 9:1 | 3.8 | 4 | -33.7 |
| 6 | 9:1 | 4.3 | 2 | -26.2 |
| 7 | 10:1 | 3.3 | 4 | -25.26 |
| 8 | 10:1 | 3.8 | 2 | -9.34 |
| 9 | 10:1 | 4.3 | 3 | -36.9 |
| Average 1 | -25.333 | -19.837 | -14.197 |  |
| Average 2 | -29.033 | -26.463 | -33.483 |  |
| Average 3 | -23.833 | -31.900 | -30.520 |  |
| R | 5.200 | 12.063 | 19.286 |  |
| Primary and secondary factors |  | C＞B＞A |  |  |
| Optimal solution |  | C2 B3A2 |  |  |

Table S6 Characteristic FT-IR peaks present in the HAA composite nanoparticles

|  | Functional groups | Wavenumber（cm-1） |
| --- | --- | --- |
| ATC | O-H stretching vibration | 3382 |
|  | C=O stretching vibration | 1641 |
|  | C=C stretching vibration | 1444 |
|  | C-O angular deformations of phenol | 1326 |
|  | Benzopyran aromatic ring stretching vibration | 1245 |
| HA | O-H stretching vibration | 3459 |
|  | C-H stretching vibration | 2918 |
|  | Amide Ⅰ (C=O stretching) | 1633 |
|  | COO- stretching vibration | 1417 |
|  | C-O stretching | 1046 |
| HAA | C=O stretching | 1648 |
|  | COO- stretching vibration | 1409 |
|  | C-O angular deformations of phenol | 1303 |
|  | C-O stretching | 1049 |
